# Comparison of continuous measures across diagnostic PD-L1 assays in non-small cell lung cancer using automated image analysis

**DOI:** 10.1038/s41379-019-0349-y

**Published:** 2019-09-16

**Authors:** Moritz Widmaier, Tobias Wiestler, Jill Walker, Craig Barker, Marietta L. Scott, Farzad Sekhavati, Alexei Budco, Katrin Schneider, Felix J. Segerer, Keith Steele, Marlon C. Rebelatto

**Affiliations:** 1 Definiens AG, Munich, Germany; 2 Oncology Companion Diagnostics Unit, Precision Medicine and Genomics, IMED Biotech Unit, AstraZeneca, Cambridge, UK; 3 Diagnostic Development Unit, Precision Medicine, R&D Oncology, AstraZeneca, Cambridge, UK; 4 MedImmune, Gaithersburg, MD USA

**Keywords:** Diagnostic markers, Non-small-cell lung cancer, Diagnostic markers, Non-small-cell lung cancer

## Abstract

Tumor programmed cell death ligand-1 (PD-L1) expression is a key biomarker to identify patients with non-small cell lung cancer who may have an enhanced response to anti-programmed cell death-1 (PD-1)/PD-L1 treatment. Such treatments are used in conjunction with PD-L1 diagnostic immunohistochemistry assays. We developed a computer-aided automated image analysis with customized PD-L1 scoring algorithm that was evaluated via correlation with manual pathologist scores and used to determine comparability across PD-L1 immunohistochemistry assays. The image analysis scoring algorithm was developed to quantify the percentage of PD-L1 positive tumor cells on scans of whole-slide images of archival tumor samples from commercially available non-small cell lung cancer cases, stained with four immunohistochemistry PD-L1 assays (Ventana SP263 and SP142 and Dako 22C3 and 28-8). The scans were co-registered and tumor and exclusion annotations aligned to ensure that analysis of each case was restricted to comparable tissue areas. Reference pathologist scores were available from previous studies. F1, a statistical measure of precision and recall, and overall percentage agreement scores were used to assess concordance between pathologist and image analysis scores and between immunohistochemistry assays. In total, 471 PD-L1-evalulable samples were amenable to image analysis scoring. Image analysis and pathologist scores were highly concordant, with F1 scores ranging from 0.8 to 0.9 across varying matched PD-L1 cutoffs. Based on F1 and overall percentage agreement scores (both manual and image analysis scoring), the Ventana SP263 and Dako 28-8 and 22C3 assays were concordant across a broad range of cutoffs; however, the Ventana SP142 assay showed very different characteristics. In summary, a novel automated image analysis scoring algorithm was developed that was highly correlated with pathologist scores. The algorithm permitted quantitative comparison of existing PD-L1 diagnostic assays, confirming previous findings that indicate a high concordance between the Ventana SP263 and Dako 22C3 and 28-8 PD-L1 immunohistochemistry assays.

## Introduction

Tumors can evade the immune system via exploitation of inhibitory checkpoint pathways that suppress antitumor T-cell responses [1]. In the programmed cell death-1 (PD-1) and programmed cell death ligand-1 (PD-L1) pathway, the PD-L1 expressed by tumor or tumor-infiltrating immune cells binds to PD-1, inhibiting T-cell receptor signaling and blocking antitumor immune response [2–4]. Antibodies targeting PD-1 or PD-L1 can block this interaction, thus resuming antitumor response [2].

The introduction of immune checkpoint inhibitors has transformed the treatment landscape for several cancers, including non-small cell lung cancer. Tumor PD-L1 expression is a key biomarker to identify patients who may have an enhanced response to non-small cell lung cancer treatment using PD-1 or PD-L1 inhibitors [5–10]. Each treatment is currently used in conjunction with a Food and Drug Administration-approved individual diagnostic immunohistochemistry assay, as either a companion or complementary diagnostic test, to assess PD-L1 expression levels on malignant tumor and/or immune cells. Pembrolizumab, for example, is approved for use in metastatic non-small cell lung cancer as first-line treatment in patients with ≥ 50% of tumor cells expressing PD-L1, as determined by a Food and Drug Administration-approved test [11], such as the Dako IHC PD-L1 22C3 pharmDx companion assay used in the trial underlying approval [11, 12]. Nivolumab is approved for use in patients with metastatic non-small cell lung cancer who have progressed on/after platinum-based chemotherapy [13], with Dako PD-L1 28-8 pharmDx approved as a complementary PD-L1 diagnostic test [14]. Other commercially available PD-L1 immunohistochemistry tests include the Ventana SP142 and SP263 assays [15–17]. SP263, for example, is approved as a companion diagnostic test with durvalumab, which was recently approved in the European Union for use in locally advanced, unresectable non-small cell lung cancer in adults whose tumors express PD-L1 on ≥ 1% of tumor cells and whose disease has not progressed following platinum-based chemoradiation therapy [18]. All four assays have been developed independently with different antibody clones, immunohistochemistry protocols, scoring algorithms, and cutoffs to define high versus low PD-L1 expression levels [19–22]. However, previous studies using commercial non-small cell lung cancer tumor samples have shown strong concordance for PD-L1 tumor cell staining at different cutoffs for three of the four immunohistochemistry assays (i.e., the Ventana SP263 and Dako 22C3 and 28-8 assays), with less agreement using the Ventana SP142 assay [23–25].

Recent studies have shown that the scoring variation between different pathologists can be an intrinsic source of error [25–27]. For example, a recent study noted that the variability between different pathologists who were scoring the same stained samples appeared higher than the variability between different immunohistochemistry assays scored by a single reader, as reflected in the overall percentage agreement scores [25]. Thus, the practice of assigning a pathologist score manually may lead to inconsistent results. Automated image analysis may provide an aided scoring tool for pathologists to reduce inter- and intra-reader variability and increase scoring throughput (e.g., via a time advantage by eliminating the need for manual area selection on stained samples).

Here we report an extension of previous studies [24, 25] using archival tumor samples from commercially available non-small cell lung cancer cases in which automated image analysis with a customized PD-L1 scoring system was developed, evaluated via correlation with manual pathologist scores, and then used to determine comparability across the four PD-L1 immunohistochemistry assays, based on a more quantitative comparison. The PD-L1 scoring used in this analysis was developed to detect stained tumor cells, owing to their use in commercially available companion tests [24, 25].

## Methods

### Tumor samples and assays

**Fig. 1:**
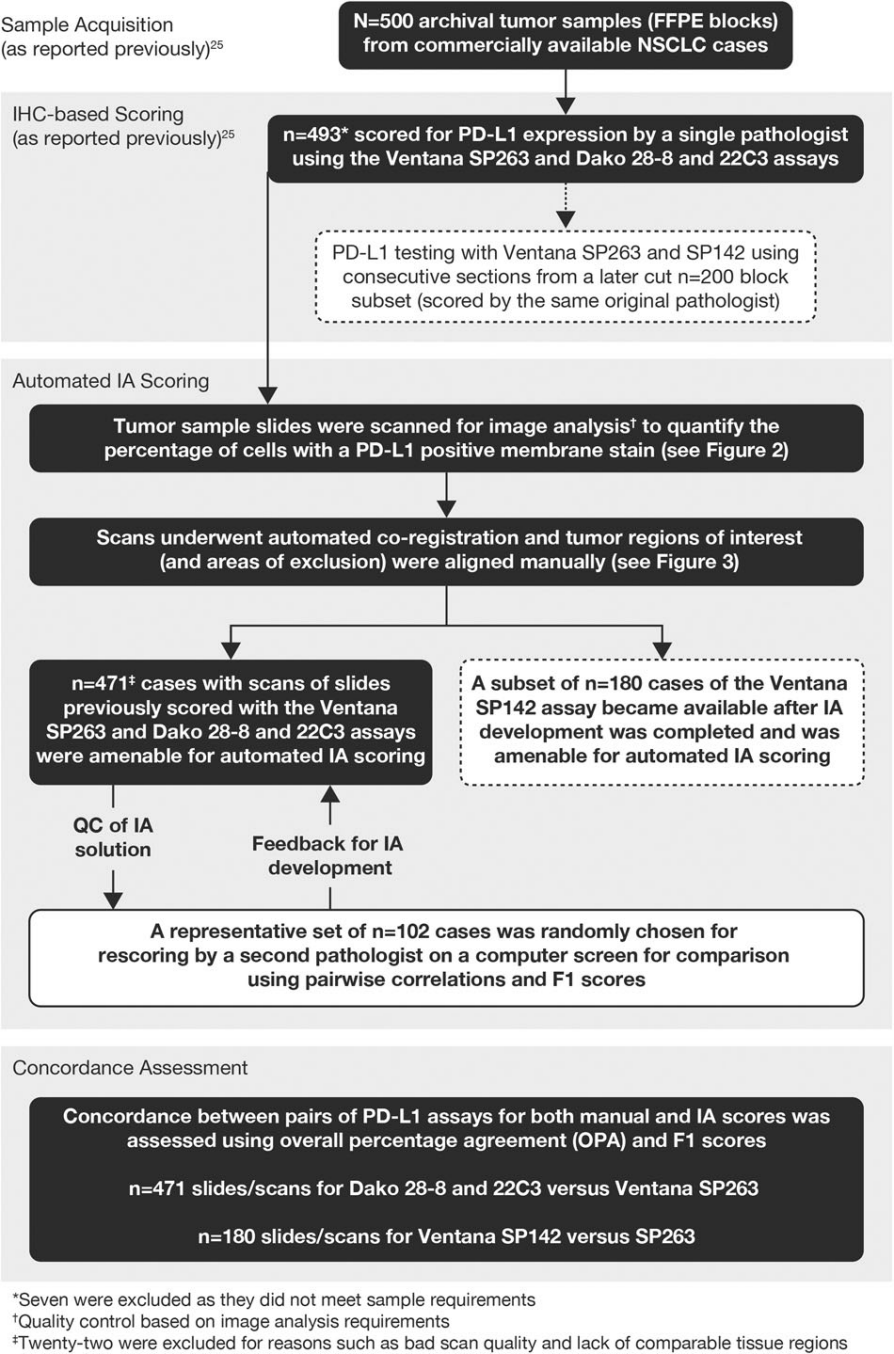
Study design with IA scoring algorithm

As reported previously [25], 500 archival tumor resection samples (formalin-fixed, paraffin-embedded blocks) were obtained from commercially available non-small cell lung cancer cases (Asterand; ProteoGenex; Tissue Solutions). Of these, 493 were evaluable for PD-L1 expression using the Ventana SP263 and Dako 28-8 and 22C3 assays and read in batches on an assay-by-assay basis by a single manufacturer-trained pathologist in a Clinical Laboratory Improvement Amendments program-certified laboratory (Hematogenix; Tinley Park, IL, USA) (Fig. 1). Using consecutive sections from a later cut (*n* = 200) block subset, PD-L1-evaluable samples were scored by the same original pathologist using the Ventana SP263 and SP142 assays (Fig. 1). Within-block concordance for repeated SP263 staining has been previously shown [28].

### Image analysis scoring algorithm

**Fig. 2:**
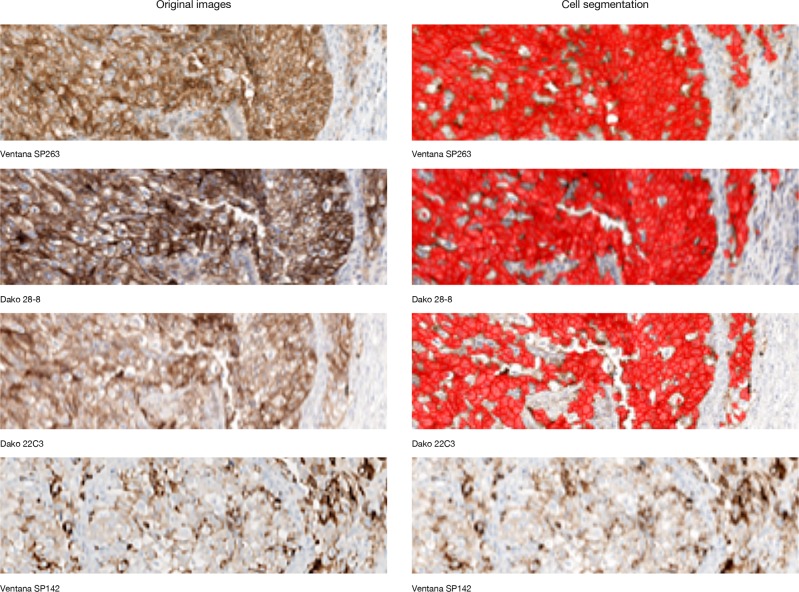
Segmentation of positive PD-L1 cells. Due to differences in intensity and presentation of stain on tumor cell membranes, automated detection of positive cells using the same (assay-independent) thresholds revealed differences between the assays

**Fig. 3:**
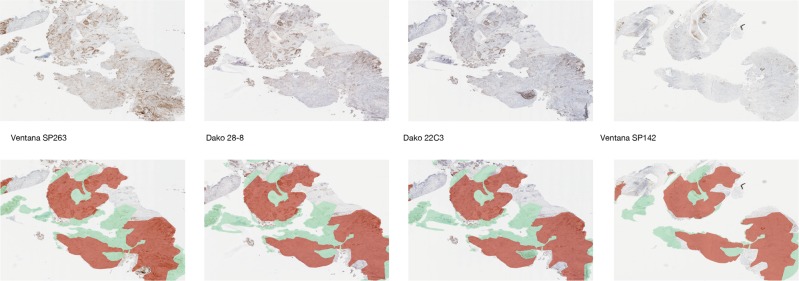
Co-registered regions of interest. Top panel: original scanned images. Bottom panel: manual exclusion of artefacts on each scan (scanner artefacts and staining artefacts) and co-registered annotation transfer (tumor center, exclusion) [30]

An image analysis scoring algorithm (Fig. 1) [29] was developed (based on the scores for 70 non-small cell lung cancer cases) to quantify the percentage of tumor (neoplastic) cells (excluding identified immune cells) with a positive membrane stain on a full-slide scanned image (“scan”) of all samples for the four PD-L1 immunohistochemistry assays (Ventana SP263, Dako 28-8, Dako 22C3, and Ventana SP142) (Figs. 1 and 2). Slides were scanned on an Aperio Scanscope scanner at 20x magnification. The scans were automatically co-registered [30] and tumor as well as exclusion annotations were aligned manually to ensure that analysis of each case was restricted to tissue areas comparable across the four assays (Figs. 1 and 3). Pathologist scores for the percentage of membrane-positive tumor cells, generated via microscope (“slides”) for the original physical samples, were available from previous studies [24, 25] and used as the reference for the immunohistochemistry assay comparisons. The areas of pathologist assessment were not annotated, but the areas analyzed comprise most of the tumor region and hence overlap with the scanned and image analysis-analyzed regions. For evaluation of the quality of the automatically generated image analysis scores, a randomly chosen subset of the scans (using the Ventana SP263 and Dako 28-8 and 22C3 assays) were rescored by a second pathologist on a computer screen (Fig. 1). In addition to being blinded to the immunohistochemistry assay, the second pathologist only used comparable regions for scoring.

### Overall percentage agreement

Total agreement for positive and negative ratings between two immunohistochemistry assays was measured as overall percentage agreement [31], taking into account concordant classification of samples above and below the thresholds applied for each assay:

$${\mathrm{OPA}} = \frac{{{\mathrm{TP}} + {\mathrm{TN}}}}{{{\mathrm{All}}{\;}{\mathrm{samples}}}}$$

in which TP and TN denote “True” positive and negative, respectively.

### F1 score

The F1 score was used to measure concordance for “positive” ratings (i.e., samples above a given cutoff) between different immunohistochemistry assays or between pathologist and image analysis scores from the same immunohistochemistry assay at any given cutoff pair from 1 to 99% positive cells (in 1% increments) [32]:

$${\mathrm{F1}}{\;}{\mathrm{score}} = \frac{{2^\ast {\mathrm{TP}}}}{{2^\ast {\mathrm{TP}} + {\mathrm{FN}} + {\mathrm{FP}}}}$$

in which TP denotes “True” positive (i.e., both assays agree), and FP or FN denote “False” positive or negative (i.e., the classifications based on the assays disagreeing at this cutoff pair).

For comparisons of image analysis scores against pathologist scores, the pathologist ratings can be considered as “ground truth”; thus, concordant ratings based on image analysis are “True” positive and discordant ratings are “False” positive and “False” negative. Importantly, although for comparisons between pathologists either can be considered as “ground truth”, the calculation gives the same result. Following validation of the image analysis scoring algorithm, F1 was used to compare immunohistochemistry assays based on both pathologist and image analysis scores.

Unlike overall percentage agreement, which provides an intuitive score of the overall agreement in ratings, the F1 score focuses on agreement for positive patients which, in some instances, can be more important (e.g., for patient segmentation). For this analysis, both F1 and overall percentage agreement were used since they provide different types of information.

### Image analysis optimization

As an ad-hoc analysis, a new image analysis workflow was introduced that allowed dynamic (re-)assessment of tumor cell positivity by varying different thresholds. In brief, the workflow can be outlined as follows: tumor cells were segmented in a scan (regardless of the presence of stain), with its features extracted and thresholds for these features optimized to maximize correlation with the Ventana SP263 assay reference data from previous studies [24, 25] (see Section S1 in the Supplementary Methods for more details). To test the robustness of this approach and to avoid over-fitting, data from the complete dataset were split into two groups: one for training and another for testing.

## Results

### Patients and tumor samples

As reported previously, PD-L1-evaluable tumor (neoplastic) resection samples were obtained from 493 patients with Stage I–IV non-small cell lung cancer, the majority of whom (75.3%) were Caucasian and approximately half each had squamous and non-squamous histology [25]. Of the 493 slides previously rated by pathologists, 471 were amenable for automated image analysis scoring using the Ventana SP263 and Dako 28-8 and 22C3 immunohistochemistry assays (reasons for exclusion included poor scan quality and lack of comparable tissue regions) (Fig. 1). Of the *n* = 200 block subset previously scored using the Ventana SP142 assay, 180 were evaluable for image analysis (Fig. 1).

### Comparison between pathologist scores and image analysis scores

**Fig. 4:**
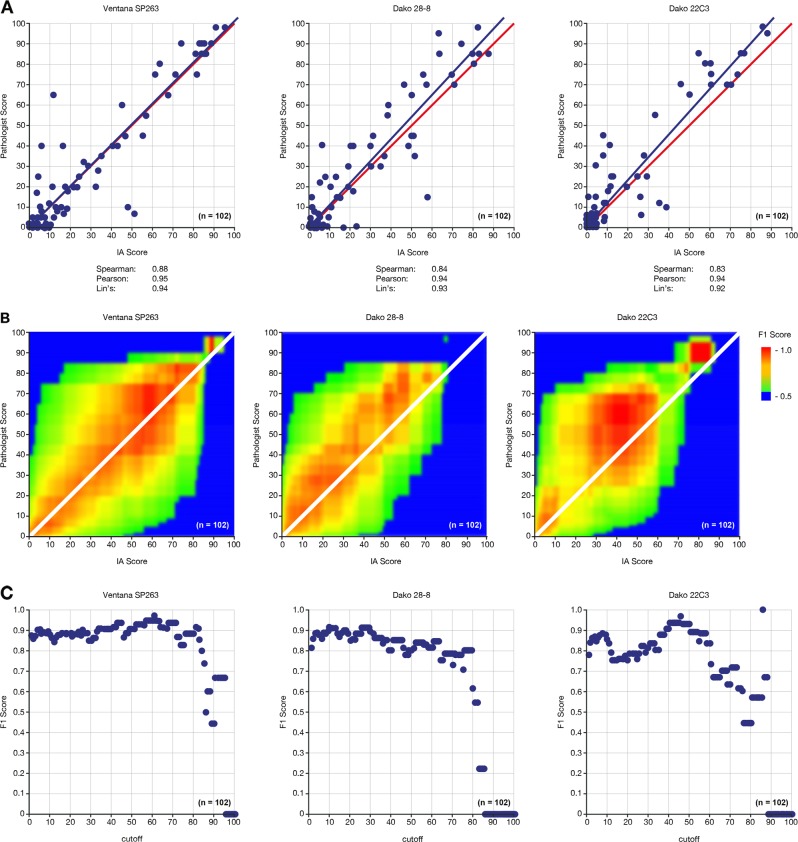
**a** Pairwise correlations, **b** F1 concordance scores, and **c** F1 scores from 1:1 matched assay cutoffs for pathologist versus IA scores. A randomly chosen subset of the scans (*n* = 102) was rescored by a second pathologist on a computer screen (blinded to the assay) using only comparable regions for scoring. In panel **a**, the blue lines denote “best fit” or linear regression lines

Using a randomly chosen subset of scans (*n* = 102) for which a second pathologist was blinded to the assays (but based on the assumption that the second pathologist’s ratings were “ground truth”), the automated image analysis achieved high to very high positive linear correlation [33] with the pathologist scores generated on the same scans and regions (e.g., Spearman and Pearson correlation coefficients were in the range of 0.83–0.88 and 0.94–0.95, respectively; Fig. 4a). The high correlation was reflected by high F1 concordance values, which center around 1:1 matched cutoff pairs (Fig. 4b). Closer examination of 1:1 matched assay cutoffs revealed reasonable F1 concordance (0.8‒0.9) for the Ventana SP263 and Dako 28-8 immunohistochemistry assays for cutoffs of 3% up to 65% (Fig. 4c). For Dako 22C3, a similar but slightly inferior profile was observed, with comparably low concordance around the 20% threshold. Generally, for lower (and even more so for higher) cutoff pairs, concordance was reduced. The latter is at least partially explained by the low number of strongly positive cases and slightly lower sensitivity of the automated image analysis (see the linear regression fit line in Fig. 4a). Similar results, with high linear correlation and F1 concordance, were observed when comparing the automated image analysis with mean readout scores from the 2 pathologists (Fig. S1); high correlation was also observed between the image analysis score and each pathologist independently.

### Overall percentage agreement between assays

**Fig. 5:**
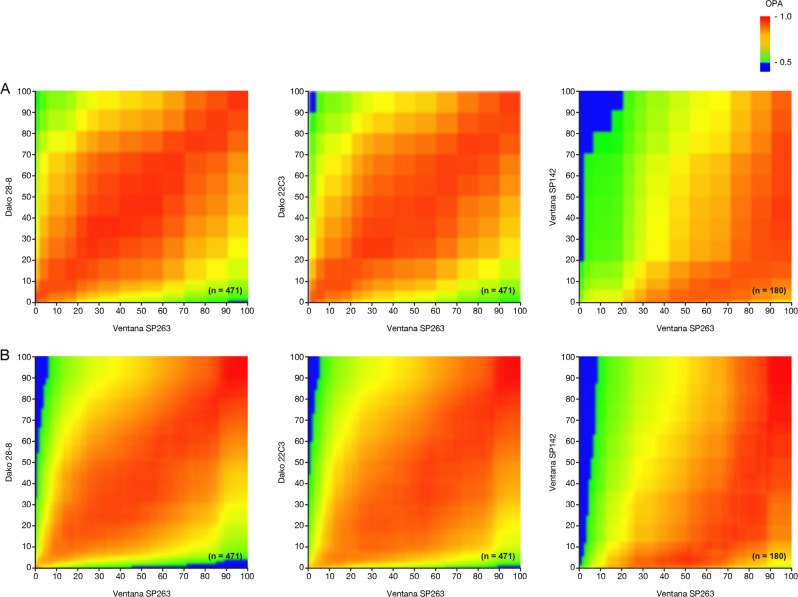
OPA scores between pairs of assays for **a** pathologist scores [24, 25] and **b** IA scores

In the overall percentage agreement calculations, the manual scores from previous publications based on slides [24, 25] (Fig. 5a) and the newly produced image analysis scores based on scans (Fig. 5b) displayed very similar results, further confirming the previous results and, indirectly, the quality of the image analysis results. Consistently, the automated image analysis scores displayed similar results to the mean readout scores from the 2 pathologists (Fig. S2).

### F1 scores between assays

**Fig. 6:**
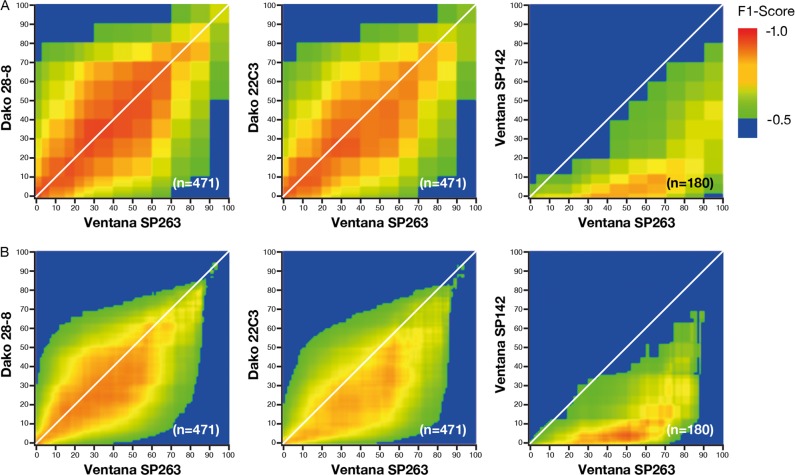
F1 scores between pairs of assays for **a** pathologist scores [25] and **b** IA scores

In this dataset, the numbers of PD-L1 negative (below threshold) samples exceeded the number of PD-L1 positive samples at most cutoffs; thus, overall percentage agreement scores were largely driven by the concordance of negative samples. F1 scores, however, restrict the comparisons to the more relevant, albeit smaller, group of positive samples. Both overall percentage agreement and F1 scoring showed concordance between Ventana SP263, Dako 28-8, and Dako 22C3 immunohistochemistry assays; however, Ventana SP142 showed very different characteristics (Fig. 6). Small differences between the previous manual scores (Fig. 6a) and automated scores (Fig. 6b) were observed. Compared with the human pathologist, the automated image analysis of the Dako 22C3 immunohistochemistry assay identified slightly lower percentages of positive cells, as evident in the slight skew in the F1 plot (Fig. 6b).

### Image analysis optimization

**Fig. 7:**
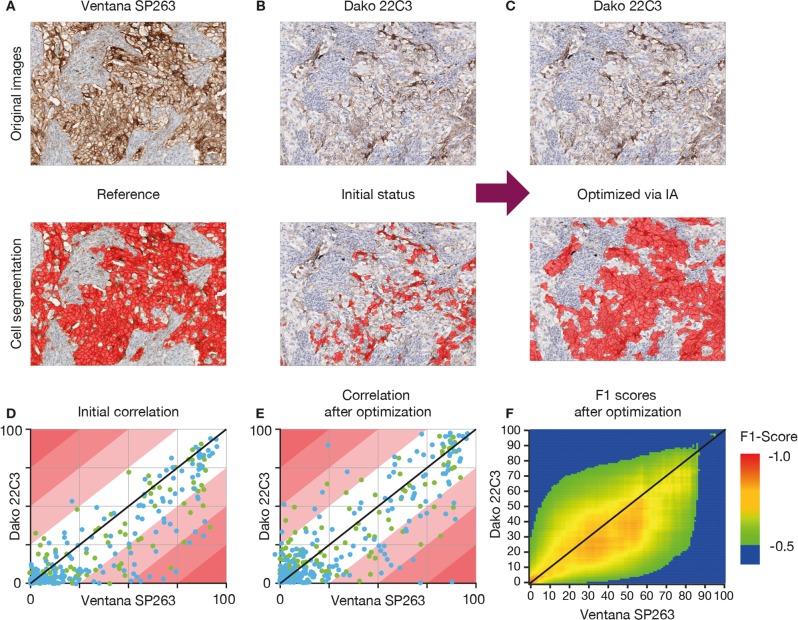
IA optimization of positivity thresholds for tumor cells. In panels **d** and **e**, the points of the plotline falling on the diagonal line indicate a perfect match of the pathologist and IA scores. The red lines/regions indicate regions of increasing discordance between both ratings in 25% (absolute value) increments (e.g., the white region indicates a region where both ratings agree by ±25% and the light red region is where both ratings agree by ±50%, etc.). In panel **f** (after optimization), the skewness is removed (perhaps, however, at the cost of slightly inferior overall accuracy)

As an ad-hoc analysis, a new image analysis workflow was introduced (see Section S1 in the Supplementary Methods) that allowed dynamic (re-)assessment of tumor cell positivity by varying different thresholds (Fig. 7a, b). An optimal set of thresholds was identified by iterating through thresholds for Dako 22C3 and comparing the results against the established image analysis scores from Ventana SP263 (Fig. 7c). Using the complete dataset (*n* = 470), split into training and testing groups, two immunohistochemistry-related features and their thresholds were identified that yielded the best results: a ratio membrane to overall diaminobenzidine intensity of  >1.02 and a mean overall diaminobenzidine intensity in the membrane of  >17.72. A linear correlation between both assays (initial [Fig. 7d] and after optimization [Fig. 7e]) was reached that eliminated the skewness from the F1 plot (Fig. 7f).

## Discussion

We showed that a novel automated image analysis scoring algorithm can be used to determine tumor cell PD-L1 positivity in patients with non-small cell lung cancer and that it demonstrates high analytical concordance with pathologist ratings, permitting quantitative comparison of existing immunohistochemistry assays in order to confirm previous findings [25]. Both the manual and automated image analysis approaches showed that the Ventana SP263, Dako 28-8, and Dako 22C3 assays are highly concordant for a broad range of cutoffs on an analytical level, as reflected in both overall percentage agreement and F1 scoring, while the Ventana SP142 assay showed very different characteristics. The reasons for the distinct profile for SP142 may stem from different optimization of the assays, as well as a lower titration of SP142 compared to the other assays; these outcomes are consistent with numerous other studies showing divergent outcomes when comparing SP142 staining to other assays [23, 34, 35]. While the SP142 was not compared directly with the Dako 28-8 or Dako 22C3 assays, the high concordance of these two assays with SP263, in parallel with the low concordance between SP142 and SP263, suggest that SP142 would also show lower outcome concordance with the other two assays. The F1 scores highlight that the automated image analysis of Dako 22C3 identified slightly lower percentages of PD-L1 positive tumor cells than did pathologists. Importantly, differences between immunohistochemistry assays may have been surmounted by human translational capabilities in the previous study [25] (e.g., human pathologists were required to “interpret” subtle differences in staining presentation between assays for which this algorithm is not tuned). In comparison to previous efforts based on pathologist ratings, no assay-specific adaptations based on interpretation were made by our algorithm (i.e., independent of the immunohistochemistry assay, each cell was tested against the same criteria for positivity). However, it was demonstrated that inter-assay differences can be minimized by optimization of image analysis morphology- and immunohistochemistry-related parameters and their thresholds, indicating that this could be used to improve consistency and concordance with pathologist ratings or other relevant measures (e.g., outcome and gene or protein expression).

Response rates to anti-PD-1 and anti-PD-L1 agents have been shown to be greater in patients whose tumors express high levels of PD-L1 compared with those expressing low or no tumor PD-L1 [1–4]. Broad access to high-quality PD-L1 testing will help clinicians to identify the most appropriate treatment option for individual patients. High concordance between most diagnostic tests has been reported earlier [23, 25]. In the recent study by Ratcliffe et al. the variability between two different pathologists scoring the same stained samples appeared higher than the variability between different assays scored by a single reader; in addition, concordance between different pathologists scoring the same slides was lower for samples with staining below 10% [25]. In the case of image analysis scoring, inter-reader variability can be reduced using digitized scoring and optimization, ensuring consistent and reproducible readouts across the board.

Our results, showing the ability of automated image analysis to assess PD-L1 status in patients with non-small cell lung cancer, complement those of a recent analysis using data from a Phase 1/2 study, which demonstrated that an automated image analysis signature (based on combined baseline cell densities of PD-L1[ + ] tumor cells and CD8[ + ] tumor infiltrating lymphocytes) may allow better identification of responders to durvalumab monotherapy compared with manual PD-L1 scoring alone [36]. Furthermore, in a separate study that employed a deep semi-supervised and generative learning network, automated PD-L1 scoring of non-small cell lung cancer tumor tissue needle biopsies was concordant with visual scoring by pathologists [37]. It is important to note that, using a PD-L1 staining assay, pathologists occasionally fail to distinguish between tumor-infiltrating PD-L1 + macrophages from PD-L1 + tumor cells. As such, inclusion of macrophages in the image analysis-generated PD-L1 assessment may in fact lead to more reproducible outcomes. However, the concordance across the different assays (particularly Ventana SP263, Dako-22C3, and Dako 28-8), as well as between the pathologist and image analysis analyses, suggests that the impact of PD-L1 + macrophages on assay readout is minimal. As such, the scoring algorithms used in non-small cell lung cancer do not account for macrophages. Novel multiplexing assays using immunofluorescence to identify PD-L1 + macrophages are of interest and would provide greater insight into the immune-context of tumors.

A potential limitation of an image analysis approach is that scans cannot represent all of the details that are visible under a microscope (especially at higher resolution); hence, a deviation in results to a certain extent is to be expected [38]. In addition, while automated image analysis is expected to deliver results with low variance even in the low positivity range, it remains to be demonstrated if those results are also accurate. Comparison to ratings of multiple pathologists is required to validate this further.

**Fig. 8:**
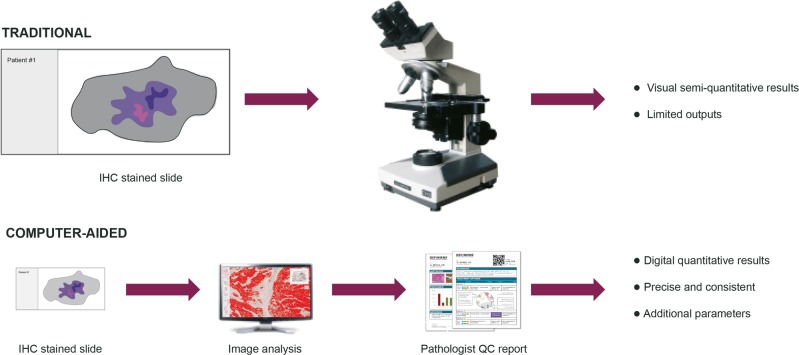
Automated IA as an aided scoring tool

In summary, the digital quantitative results from automated image analysis scoring demonstrated comparable accuracy and consistency to that provided by pathologists’ scoring. As such, image analysis scoring could serve as an aid, with appropriate validation, for PD-L1 diagnostic testing for pathologists in the clinical setting (Fig. 8) and help support scoring with commercial assays.

## Supplementary information

Supplementary Material
