# Supplementary material for: Comparison of continuous measures across diagnostic PD-L1 assays in non-small cell lung cancer using automated image analysis
Source: Mod Pathol. 2019 Sep 16;33(3):380–90. doi: 10.1038/s41379-019-0349-y (PMC7051919; doi:10.1038/s41379-019-0349-y)
Supplement: Supplementary file 1 — Supplementary Material [file 41379_2019_349_MOESM1_ESM.docx]

**Supplementary Methods**

*S1.* *Threshold optimization workflow*

In order to increase comparability of scorings between the Dako 22C3 assay and the established Ventana SP263 assay, a novel image analysis approach was applied in this study. The workflow can be outlined as follows:

- In an initial step, the image of the tissue (**Fig. S3A**) was separated into tumor (or epithelial) regions and stroma regions (**Fig. S3B**). Afterwards, all cells in the tumor area were segmented, regardless of the presence of stain (**Fig. S3C**). In this step, the programmed cell death ligand-1 (PD-L1) stain, if present, as well as the negative nuclei, are used as a basis for segmentation. For each of these cells, we stored information about its properties as values for a broad set of features. This included morphology-based features, characterizing size and shape, as well as immunohistochemistry-related features, such as the intensity or area coverage of the PD-L1 stain. Afterwards, the feature-space was sampled in the following iterative process:

1. Thresholds were set for PD-L1 positivity for the features of interest.
2. Positive cells were classified according to these thresholds (**Fig. S3D**).
3. Readouts were calculated, such as the image analysis score based on this cell classification.
4. The case-wise correlation to the reference Ventana SP263 assay, based on these readouts, was calculated.
5. Steps 1–4 were repeated for a different set of thresholds, until the parameter space was sufficiently sampled.

In this way, the correlation to the reference was evaluated as a function of the thresholds for the used features. In this study, the highest correlations between the image analysis scores for Dako 22C3 and Ventana SP263 were reached when features indicating the average intensity of the brown area within a cell were used.

**
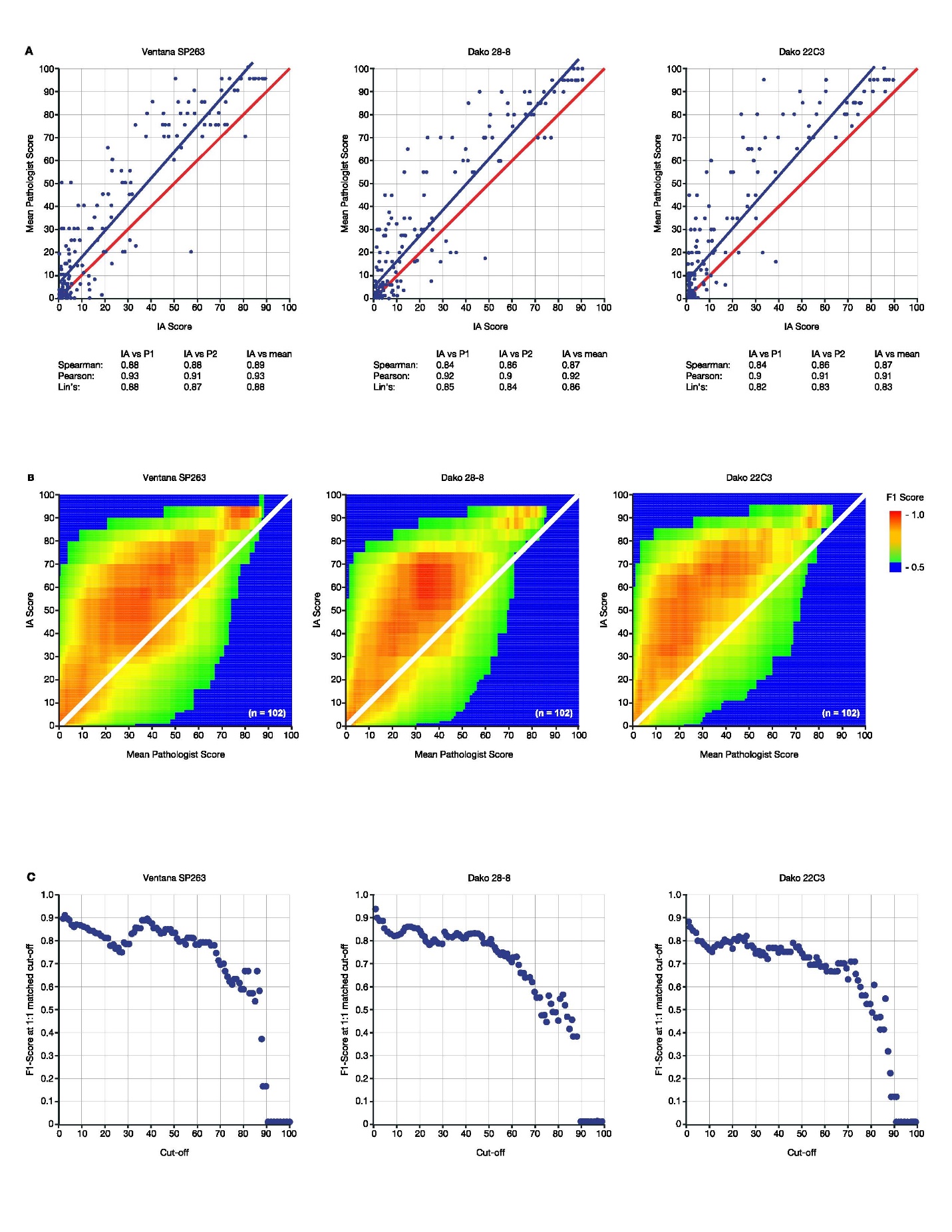
Fig. S1.** (**A**) Pairwise correlations, (**B**) F1 concordance scores, and (**C**) F1 scores from 1:1 matched assay cutoffs for image analysis (IA) versus mean pathologist scores. P1, pathologist 1; P2, pathologist 2.

**Fig. S2.** Overall percentage agreement (OPA) scores between image analysis (IA) and mean pathologist scores for the different assays.**
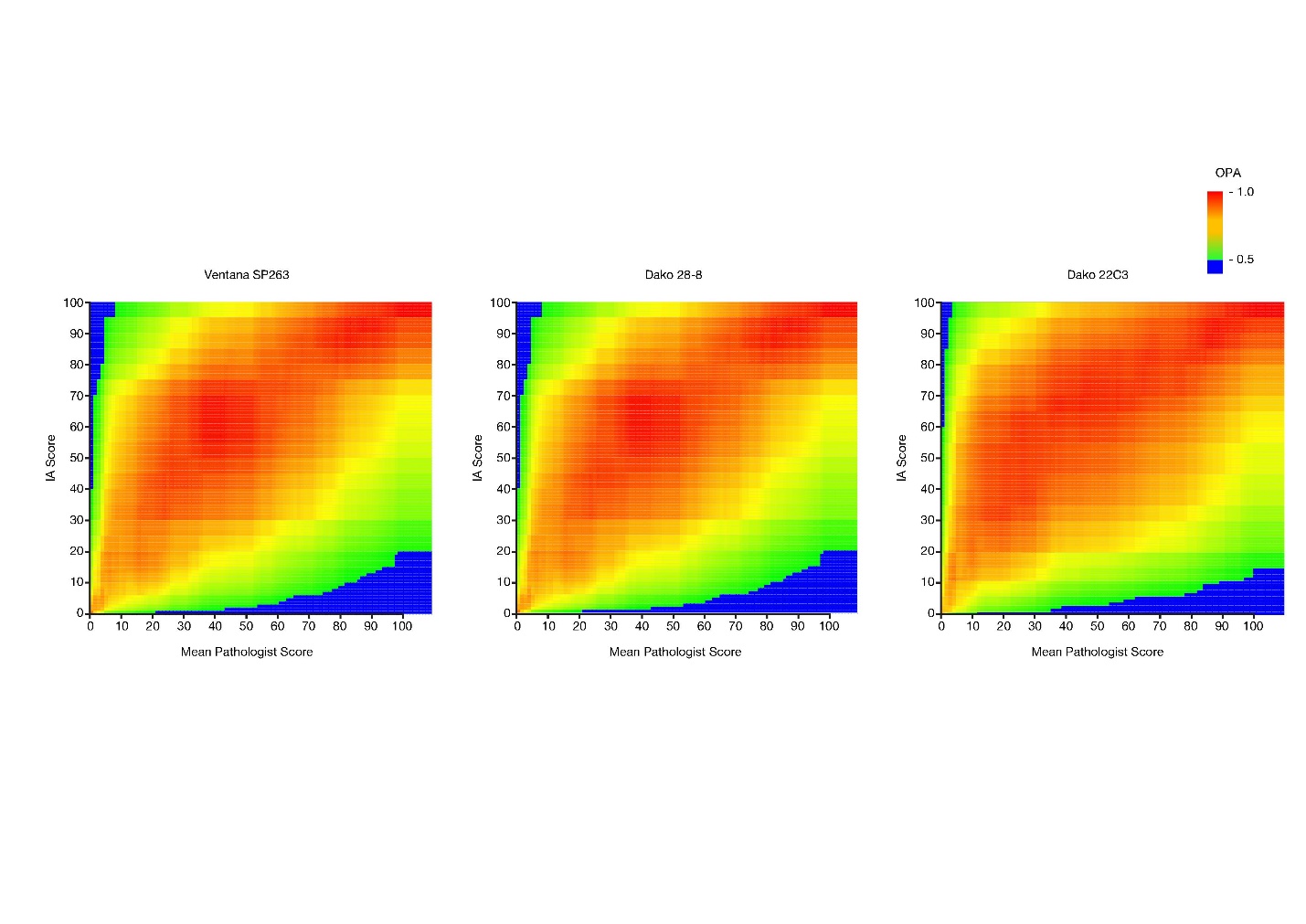
**

**Fig. S3** Threshold optimization workflow


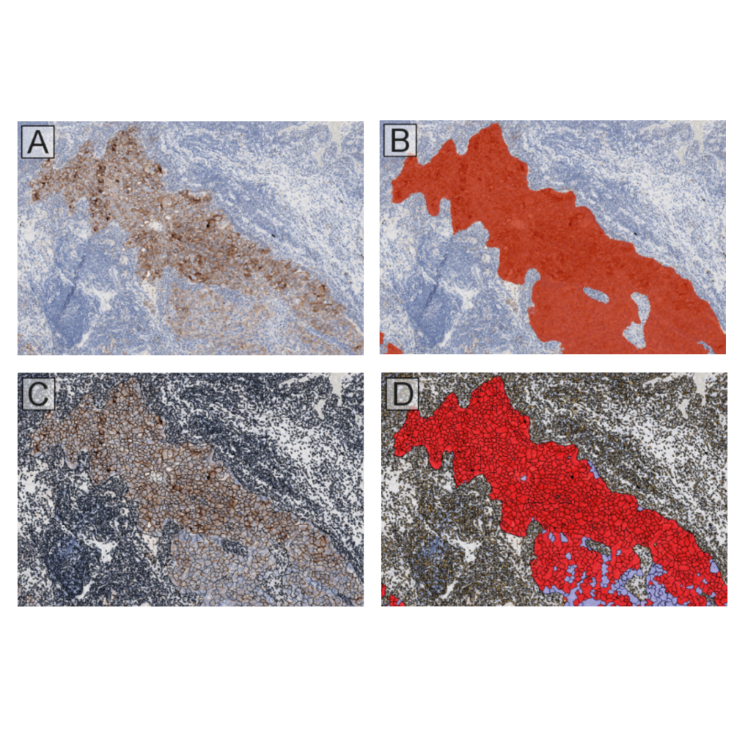
 (**A**) Original image. (**B**) Separation into epithelial (red) and stroma (no coloring) areas. (**C**) Segmentation on a cellular level. (**D**) Classification of cells into PD-L1 positive tumor cells (red) and PD-L1 negative tumor cells (blue)
